# Supplementary material for: Content-rich biological network constructed by mining PubMed abstracts
Source: BMC Bioinformatics. 2004 Oct 8;5:147. doi: 10.1186/1471-2105-5-147 (PMC528731; doi:10.1186/1471-2105-5-147)
Supplement: Additional File 5 — The original Chilibot query results of the term "long-term potentiation (LTP)" and 22 other terms, limiting the latest references analyzed to the years 1990, 1995, 2000, and 2004. [file 1471-2105-5-147-S5.bz2 › chilibotAdditionalFile5/ltp2004/html/PKC.html]

 


**PKC** (Input: PKC ) 

---


**Google Searches:** Entire Web  | EDU domain only  | PDF files only

.

**External Links:** OMIM |
LocusLink
| Swissprot
| GeneCards

  
**Maps of PKC**

Simple
Complete
graph in
radiant
tree
square
layout.

**New Hypothesis !**

**Synonyms** 

- pkc   [PubMed]

**Synopsis**

- These results suggest that activation of CaMKII, PKA and **PKC** may be crucial for the induction and the early phase  J NeurophysiolJ Neurophysiol, 2003 
  [23]

- In our system, calcineurin CaN played an essential role, rather than the activation of protein kinase C **PKC** as documented in the conventional model.  Neural Netw, 2003 
  [21]

- These results suggest that both tetanically induced and phorbol ester induced LTP, in the rat, share similar mechanisms which involve, at least in part, activation of **PKC** dependent mechanisms to increase quantal release from sympathetic preganglionic axon terminals.  Auton Neurosci, 2003 
  [20]

- Further, these splice variants have been shown to impart the highest cell surface expression, **PKC** potentiation and calcium kinetics to NMDAR complexes.  Brain Res Mol Brain Res, 2003 
  [19]

- Phosphorylation of RC3 neurogranin Ng by protein kinase C **PKC** is decreased in CA1 in response to H 2 O 2 treatment, irrespective of age.  J Neurosci Res, 2002 
  [19]

- Polyphosphoinositides are suggested to play a role in activating **PKC** mediated phosphorylation of SI in nerve terminals.  FEBS Lett, 1991 
  [19]

- Previous studies have shown DBA 2 mice to exhibit poor spatial contextual learning, impaired hippocampal LTP, and hippocampal mossy fiber hypoplasia, as well as reduced hippocampal **PKC** activity and expression relative to C57BL 6 mice.  J Neurochem, 2003 
  [16]

- We suggest that the physiological stimulation of mGluRs by the preconditioning stimulation produces a **PKC** dependent inactivation of subsequent group I mGluR functioning and thereby an inhibition of induction of group I mGluR dependent LTD DP induction.  Neuroscience, 2004 
  [16]

- These studies confirmed that GluR2 is modified in response to the activation of **PKC** and suggests that phosphorylation of the ubiquitous GluR2 subunit may be important in the regulation of excitatory synaptic transmission.  Neuropharmacology, 2001 
  [16]

- From these results, NAP 22 is confirmed to be distributed in the synaptic region of the spinal cord and is involved in the synaptic function relating to **PKC**.  Brain Res, 1999 
  [14]

- Compatible with this hypothesis, inherited AChE R overexpression in transgenic mice resulted in perikaryal clusters enriched with PKCbetaII, accompanied by **PKC** augmented LTP enhancement.  Mol PsychiatryMol Psychiatry, 1999 
  [14]

- **PKC** was activated through the metabotropic glutamate receptor in the hippocampal neurons.  J Biol Chem, 1992 
  [14]

- **PKC** was identified as the kinase, and PP1 and PP2B as the phosphatases responsible for regulating Ser 313 phosphorylation.  J Neurochem, 2003 
  [14]

- Our data support a pathway linking class I mGluRs with **PKC** and src to enhance the open probability of the NMDAR channel.  Physiol Behav, 2001 
  [14]

- Therefore, the activation and downregulation of distinct **PKC** isoforms may participate in the induction and maintenance mechanisms of LTD.  Brain Res Mol Brain Res, 2001 
  [12]
